# Barriers and Breakthroughs in U.S. Swine Biosecurity: A Key Informant Exploration of the Role of Human Behavior in Enhanced Biosecurity Compliance

**DOI:** 10.1155/tbed/5537668

**Published:** 2026-04-16

**Authors:** Benti D. Gelalcha, Maurine C. Chepkwony, Oluwaseun Akinyede, Moumita Das, Colin Yoder, Andres Perez, Maria Sol Perez Aguirreburualde, Cesar Corzo, Dennis N. Makau, Michael W. Mahero

**Affiliations:** ^1^ Department of Biomedical and Diagnostic Sciences, College of Veterinary Medicine, University of Tennessee, Knoxville, USA, tennessee.edu; ^2^ Center for Animal Health and Food Safety, University of Minnesota, Saint Paul, USA, umn.edu; ^3^ Department of Veterinary Population Medicine, College of Veterinary Medicine, University of Minnesota, Saint Paul, USA, umn.edu

**Keywords:** biosecurity barriers, critical control points, disease prevention, economic constraints, farm infrastructure, human behavior, swine biosecurity, text mining

## Abstract

Biosecurity measures are the main defense against infectious disease spread in swine farms. Farmers often acknowledge the need for enhanced biosecurity, but a gap remains between claiming measures and practicing them on the farm. This study looks at barriers to implementing biosecurity, key control points, and successes in Midwest U.S. swine operations. Semi‐structured interviews were held with industry experts knowledgeable about swine farms and responsible for biosecurity compliance. The interview transcripts were analyzed using qualitative text mining methods like word frequency, n‐gram analysis, topic modeling (latent Dirichlet allocation [LDA]), and thematic analysis. Seven major themes that converged with the results of content analysis emerged, which demonstrated major barriers, potential critical control points, and success stories in biosecurity compliance. Biosecurity practices differed significantly between operation types, with stricter practices in sow and boar studs compared to grow‐finish farm operations. Human behavior was identified as both the primary vulnerability and the greatest opportunity for improvement. Compliance was influenced by communication strategies, training, and employee workload levels. Limitations on barn infrastructure design, financial constraints, and risk perception were identified as further challenges to biosecurity implementation. Critical control points, such as transportation (trucks and trailers), equipment sharing, manure handling, and interactions with packing plants and cull markets, appear to be high‐risk nodes for disease transmission. Despite these challenges, success stories such as long‐term PRRS prevention through air filtration, strategic application of basic biosecurity measures, and the use of dedicated biosecurity managers have proven successful in reducing disease risks. Implementing biosecurity in swine farms is a complex system‐level challenge influenced by farm design, human, economic, and external interface factors. Thus, progress requires a comprehensive approach that combines infrastructure upgrades, risk‐based decision making, tailored training, and ongoing communication among stakeholders. This study identifies vulnerabilities and proven successes and offers practical insights to inform a more resilient biosecurity system.

## 1. Introduction

The global swine industry faces unprecedented challenges from emerging and re‐emerging infectious diseases that threaten both economic stability and food security. The global emergence, reemergence, and spread of transboundary animal diseases, such as African swine fever (ASF) and endemic diseases, including porcine reproductive and respiratory syndrome virus (PRRSV), porcine epidemic diarrhea virus (PEDV**),** have led to a heightened awareness of the critical importance of pig health and its strong relationship with biosecurity [1–4]. An ASF incursion into the U.S. swine industry has been projected to cost approximately 80 billion USD in losses [5]. Thus, effective biosecurity measures are crucial in protecting animal health against these challenging‐to‐control infectious diseases.

Biosecurity, which constitutes activities aimed at preventing the introduction and spread of disease within and between groups of animals, is a key operational component of modern swine production [3, 6]. The U.S. swine industry has increasingly acknowledged the significance of enhanced biosecurity protocols [3, 7] in protecting individual operations, maintaining market access, and sustaining public trust in pork product safety and quality [7, 8]. Despite growing awareness of biosecurity’s significance, considerable variability and inconsistencies remain in the adoption and implementation of recommended practices across farms and regions [3, 7, 9].

A recent study highlights that although producers claim to have biosecurity measures in place, there are notable gaps between perceived preparedness and actual practice, especially when evaluated against established guidelines [9]. The consequences of this implementation gap are concerning, as small biosecurity failures in individual operations can amplify major disease transmission and destabilize the industry. The disconnect between perceived readiness and actual practice may be related to various constraints, such as economic challenges, lack of awareness, or other farm‐level factors as previously reported [9–11]. However, a comprehensive understanding of these constraints and how they affect the real‐world swine production setting, and their influence on biosecurity outcomes, remains incomplete.

Understanding of barriers preventing biosecurity implementation is vital for developing targeted interventions that enhance disease prevention in the swine industry. Addressing biosecurity implementation barriers helps prevent disease transmission and ensure agricultural sustainability and food security. Furthermore, enhanced biosecurity implementation helps reduce reliance on antimicrobials, improve animal welfare standards and improve consumer trust in pork products [3, 12–15].

The modern biosecurity challenges demand research methods that reveal multifaceted implementation barriers and identify practical solutions to address real‐world swine producer constraints. Qualitative research approaches that directly involve biosecurity‐implementation stakeholders, may offer valuable insights into practical challenges and opportunities for improvement that may not be apparent via quantitative assessments. Against this backdrop, this qualitative study explores the gap between perception and practice in swine biosecurity, focusing primarily on the Midwest United States. Through key informant interviews, the study aims to gain deeper insight from experienced stakeholders and to clarify the results of producer surveys. These surveys have shown that while most farmers acknowledge the necessity of enhanced biosecurity and close to half report having some measures in place, more detailed investigation reveals a mismatch between confidence in biosecurity and the realities of on‐farm disease management. By investigating these issues, this research seeks to illuminate the factors contributing to the observed gap between belief and action in biosecurity, ultimately supporting the development of more effective strategies for disease prevention within the U.S. swine industry.

## 2. Materials and Methods

### 2.1. Study Context

Swine production significantly contributes to the United States agricultural economy, and it operates through an industrialized and specialized system [16, 17]. The U.S. swine industry operates through three primary production systems, which include farrow‐to‐finish, farrow‐to‐wean, and wean‐to‐finish [18]. The farrow‐to‐finish system, where pigs were bred, born, and raised to market weight on the same farm, used to be the dominant production type [18, 19]. Modern production has adopted multisite and contract‐based systems to enhance efficiency, biosecurity, and disease control. In these systems, the different production stages, such as breeding, nursery, and finishing, operate at separate facilities often through vertically integrated companies and coordinated producer networks [18, 20].

The largest swine production takes place in the Midwest region, with Iowa, Minnesota, North Carolina, and Illinois ranking as the top‐producing states [21]. Progressively, the industry has evolved to achieve higher productivity through advances in genetics, nutrition, housing, and disease management systems, which support both domestic market needs and export success. Despite its success, the industry continues to grapple with multiple challenges [22]. These include welfare problems, environmental sustainability concerns, antibiotic usage, outbreaks of endemic diseases, and threats of foreign animal diseases [3, 22, 23]. These concerns have led to a growing interest in precision livestock farming and enhanced biosecurity practices that support resilient production [1, 20].

### 2.2. Positionality and Reflexivity

Positionality describes the lens through which the researcher sees the world and thus their identity in relation to the study and its social and political context. It influences the data generation and analysis approach and thus is foundational to any qualitative research [24]. In this study, all researchers are veterinarians with graduate training in epidemiology and infectious disease management. Four of the authors have professional knowledge of swine production and have been intricately involved in biosecurity and disease management efforts in the swine industry. The first author (Benti D. Gelalcha) led data analysis and manuscript writing in close collaboration with the final author, Michael W. Mahero. Benti D. Gelalcha is a male, early/mid‐career researcher who has focused his work mainly on the management of infectious diseases and innovative strategies to enhance biosecurity on farms. As a veterinarian, Benti D. Gelalcha has interacted with a variety of livestock producers in different contexts and understands the role of human behavior on animal health and production, as well as the complex welfare and production decisions producers navigate. Thus, Benti D. Gelalcha has areas of sociocultural alignment and divergence with the backgrounds of the participants. The alignment stems from the fact that, like the participants, Benti D. Gelalcha is a veterinarian with 6 years of experience working with livestock producers in the U.S. to improve animal health outcomes at farm level. Conversely, the divergence arises from the fact that Benti D. Gelalcha has not directly worked with swine production systems or swine producers. However, his prior experience working with farmers across various settings, including U.S. dairy farms and international livestock production systems, provides him with a broad understanding of the barriers and constraints common across livestock production systems. Additionally, he draws upon the collective experience of co‐authors who are swine veterinarians or have extensive experience working with swine or swine production systems.

### 2.3. Theoretical Framework

We used a multi‐theory approach, which combined the transtheoretical model (TTM) of behavior change and the theory of planned behavior (TPB). The TTM model posits that behavior change occurs through a series of five distinct stages, pre‐contemplation, contemplation, preparation, action, and maintenance [25, 26]. It, therefore, effectively characterizes producers’ willingness to adopt biosecurity measures and overcome prevailing barriers by placing them into one of these five categories. This theory describes barriers to behavior change as being context and stage‐specific and therefore best dealt with using stage‐specific tailored messaging and interventions. While the TPB addresses one’s attitudes and beliefs about a behavior and how these attitudes facilitate (or hinder) action, the TTM model helped us frame questions that allowed the research team to distinguish profiles of swine producers and identify where they fell along the five stages of change. In turn, the TPB guided our evaluation of key informant data, enabling us to describe the specific attitudes and beliefs associated with producer action or inaction at each stage [25].

### 2.4. Study Design and Participant Selection

We conducted this constructivist descriptive qualitative study as part of a larger sequential explanatory mixed‐method study on knowledge, perception, and practice of swine biosecurity [27]. Data was collected through semi‐structured in‐depth interviews with key informants to explore perceptions, practices, and challenges related to biosecurity implementation on swine farms. The sampling method involved the purposive selection of key informants who had direct knowledge or oversight responsibilities for swine farm biosecurity on large commercial farms, primarily farm veterinarians, veterinary consultants, and farm managers who had combined experience of over 169 years and a median experience of 25 years (ranging from 16 to 32 years) in the swine industry. Eight key informants were interviewed. The selection criteria for participants included their expertise, involvement in biosecurity implementation, understanding of the U.S. swine production system, and willingness to participate. The study team consisted of five scholars with varying veterinary backgrounds, led by a researcher with deep expertise in preventive veterinary medicine and qualitative research methods.

This study involved representatives from six large commercial swine production companies operating across the U.S. Midwest. Collectively, these companies operate in seven Midwestern states (Minnesota, Iowa, Nebraska, Illinois, South Dakota, Indiana, and Wisconsin). The participating companies manage a wide range of production sites from approximately 100 to 1500 farms and oversee sow herds ranging between 50,000 and 250,000 sows per company. These operations are large‐scale, commercial entities that market up to 3.6 million hogs annually, a significant portion of the U.S. pork supply chain.

### 2.5. Study Guide

The interview guide was developed using a multi‐theoretical framework combining the TTM of behavior change and the TPB. The guide was designed to be similar and complementary to the questions asked in our prior quantitative study of beliefs, behaviors, and practices of farm biosecurity in the U.S. swine industry [27]. Facilitating further exploration of observations made from the quantitative study. Key discussion elements were centered on the producer’s perception of the feasibility of implementation of SPS Plan biosecurity practices, perceived effectiveness of SPS Plan biosecurity practices, the perceived benefit of SPS Plan biosecurity practices, and willingness to adopt SPS Plan biosecurity practices. Additionally, producers’ implementation of biosecurity practices with a focus on training and enforcement of a perimeter buffer area and a robust line of separation (LoS) was assessed. The challenges faced as well as successes encountered were noted.

### 2.6. Data Management

Verbatim transcription of the audio files was performed into Word files. Investigators reviewed the transcripts for quality checks against the audio recordings and compared them with the handwritten notes for consistency and accuracy.

### 2.7. Data Analyses

#### 2.7.1. Thematic Analysis

Qualitative analysis was conducted in close collaboration with the research assistants who interpreted the data during collection. Three researchers with expertise in qualitative methods independently reviewed at least two transcripts and developed initial codes through open descriptive coding. These codebooks were then merged into a single codebook through review, discussion, and consensus. Word files of the verbatim transcripts were imported into Dedoose (2025 Sociocultural Research Consultants, LLC), where the codebook was applied to digitally analyze the data. Using outputs from Dedoose, a table was generated that listed all codes along with excerpts from the transcripts corresponding to each code. After jointly coding two transcripts and iteratively revising the codebook, two analysts split the remaining eight transcripts for independent coding. The analysts reconvened after four transcripts each to refine into the coding framework emerging codes and re‐code as needed. Once coding was completed, the codes were grouped into categories based on content similarity, which were then further condensed into themes. Field notes from team members experienced in swine production, along with archival data reviewed earlier, were used during analysis to contextualize participants’ narratives and aid interpretation. Trustworthiness was ensured through analytic procedures, such as maintaining an audit trail of audio–visual files, memoing, reviewing field notes, consulting subject matter experts for context, providing detailed descriptions of key informant observations, and cross‐referencing findings across respondent categories.

#### 2.7.2. Content Analysis

To strengthen triangulation and enhance analytic rigor, the data were also analyzed using unsupervised machine learning techniques. Combined transcripts were imported into Python (v3.11, PSF, USA) via Jupyter Notebook. We conducted a content analysis to identify key themes discussed across interviews related to swine farm biosecurity, utilizing qualitative text mining techniques. The Natural Language Toolkit (NLTK) was used for text data preprocessing, including stop word removal, lemmatization, and tokenization, and the removal of special characters, punctuation, and numbers. The preprocessed text was then transformed into a document‐term matrix for further analysis. We applied word frequency analysis to identify the most common words (major themes) in swine biosecurity discussions in the text. N‐gram analyses (bigram and trigram) were used to capture significant multiword expressions and contextual pairings within the transcripts. A word cloud was generated to visualize the most frequently cited terms. To uncover overarching themes, we employed latent Dirichlet allocation (LDA) topic modeling with perplexity scores used to determine the number of topics that offered the best structure. Sentiment analysis was applied to the entire corpus of key informant interview transcripts to map the overall tone and attitudes of key informants toward enhanced biosecurity measures‐related topics raised in the discussion. Based on contextual clues and textual polarity, the analysis classified responses as positive, neutral, or negative. Frequencies of key categories and terms were quantified and summarized in charts. These computational analyses complemented the thematic coding, allowing the research team to compare inductively derived themes with patterns emerging from unsupervised machine learning. This triangulation enhanced the robustness and depth of findings. Findings from the text‐mining and topic‐modeling analyses were compared with inductively derived themes from the thematic analysis to assess convergence and refine interpretation.

## 3. Results

### 3.1. Word Cloud Analysis of Key Informant Interviews on Swine Biosecurity

**Figure 1:**
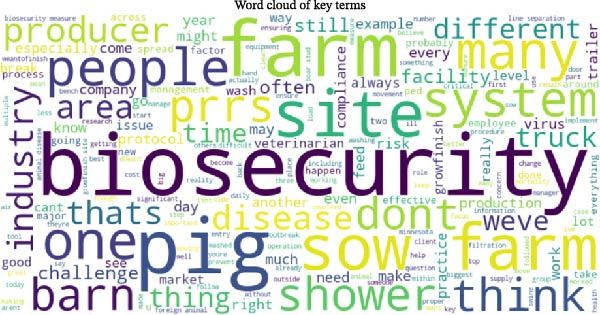
Result of word cloud visualization showing the most frequent words raised in discussion. The word cloud shows key themes of discussion related to swine biosecurity, including major topics such as biosecurity, farm, pig, PRRS, disease, sow, truck, shower, and others. The result of the word cloud confirms that biosecurity, disease prevention, farm management, and challenges to biosecurity are the main topics discussed with key informants.

A word cloud obtained from the key informant interviews transcript (Figure 1) highlighted the term “biosecurity” as the most dominant topic, followed by other frequently mentioned terms such as “pig,” “people,” “farm,” “sow,” “barn,” “site,” “producer,” and “system.” These terms reflect the core elements and stakeholders involved in applying biosecurity measures.

The frequency of the words “PRRS,” “disease,” “truck,” “protocol,” and “shower” underscores the informants’ concerns about pathogen transmission (notably PRRSV) and the importance of routine procedures (e.g., shower‐in/shower‐out) used to mitigate those risks. The word cloud (Figure 1) illustrates the most salient terms identified through the frequency and N‐gram analyses, providing an intuitive visualization of how key operational and disease‐related concepts feature prominently in informant discourse.

### 3.2. Word Frequency and N‐Gram Analysis Result

**Figure 2:**
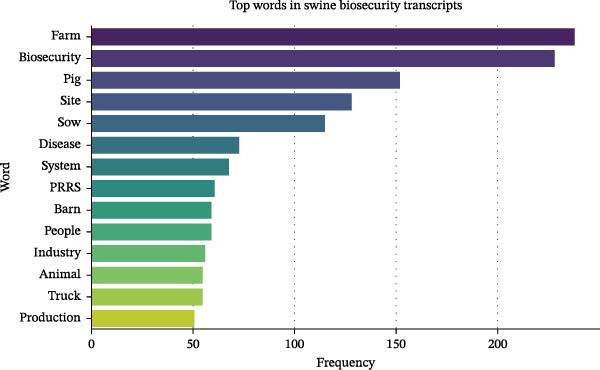
Most frequent words identified in the transcript. The most frequent words indicate a heavy focus on biosecurity in pig farming, particularly farm management, disease prevention (especially diseases like PRRS), the importance of people (employees and maintenance workers) and trucks (transporting animals and equipment) in biosecurity compliance, and the challenges of maintaining biosecurity across various farm sites, barns (structure/design), and production systems.

**Figure 3:**
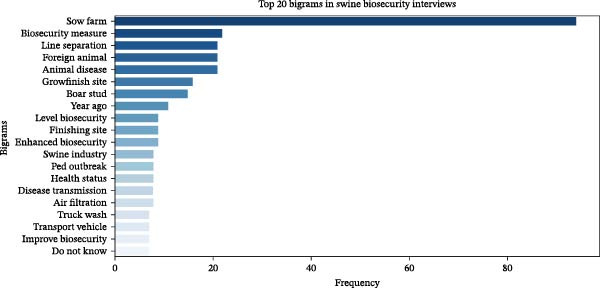
Top 20 bigrams in swine farm biosecurity assessment key informant transcript. These bigrams highlight the importance of biosecurity in various stages of pig farming, from sow farms to finishing sites, with a specific focus on practices, disease transmission, and logistical factors (e.g., truck washing). The terms reflect an emphasis on disease prevention, health management, and ensuring that measures are in place to mitigate risks, especially from foreign animal diseases.

**Figure 4:**
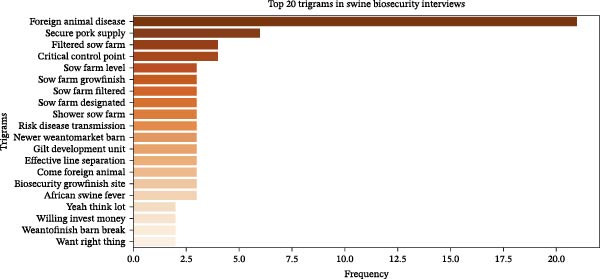
Frequency distribution for the top trigrams in swine biosecurity key informant transcripts. The trigrams, like the bigrams, highlight the critical stages and areas in swine farming where biosecurity practices are crucial for disease prevention, particularly in preventing the spread of FAD and maintaining herd health. The terms also reflect the importance of practices such as filtration, consideration of disease risks, critical control points, and the financial constraints to invest in biosecurity at different levels of production.

The frequency distribution of terms shows that “biosecurity,” “farm,” “PRRS,” “sow,” “disease,” and “truck” were the most frequently used terms that dominate the discussions in key informants’ interviews (Figure 2). Important multiword phrases identified in the transcript were found using N‐gram analysis. These included sow farm, biosecurity measures, LoS, and foreign animal disease among the bigrams (Figure 3). Trigram (3‐word phrase) analysis identified the most frequently co‐occurring sets of three consecutive words, providing deeper context into the key phrases emphasized by informants. The most common trigrams include “foreign animal disease,” “secure pork supply,” “risk disease transmission,” “critical control point,” and “filtered sow farm” (Figure 4, which corresponds to some of the key operational and behavioral risk nodes explored in the thematic analysis).

### 3.3. Topic Modeling (LDA) Analysis

A five‐topic LDA model provided the lowest perplexity score (77.92), showing the best statistical fit to the data (Table 1). In topic modeling, lower perplexity reflects a model that predicts the words in the corpus more accurately, resulting in more coherent and interpretable topics compared with other configurations. The LDA topic modeling revealed five thematically distinct yet interrelated domains in swine biosecurity adoption and management. The five identified topics capture the diversity of concerns and experiences expressed by informants, while Table 2 captures the informants’ level of contribution to these topics:

**Table 1:** Topic perplexity scores.

Number of topics	Perplexity score (lower is better)
3	82.15
4	79.87
5	**77.92**
6	78.45
7	80.12

*Note:* The bold values indicate the number of topics with the lowest perplexity score, used to determine the optimal number of topics that best describes the structure of the text or key content of the corpus without overfitting to the training text.

**Table 2:** Distribution of themes across key informants.

Key informant	Topic 1	Topic 2	Topic 3	Topic 4	Topic 5
Informant 1	0.25	0.3	0.2	0.15	0.1
Informant 2	0.22	0.28	0.18	0.2	0.12
Informant 3	0.3	0.25	0.22	0.15	0.08
Informant 4	0.2	0.32	0.18	0.22	0.08
Informant 5	0.28	0.26	0.2	0.14	0.12
Informant 6	0.18	0.22	0.24	0.26	0.1
Informant 7	0.21	0.3	0.19	0.2	0.1
Informant 8	0.26	0.27	0.18	0.19	0.1

Topic 1: Research and compliance

• Keywords: research, veterinarian, employee, company, compliance, filtration, PED, good, key, and ensuring.

Topic 2: Facility and animal management

• Keywords: barn, animal, grow‐finish, facility, health, producer, boar, stud, start, and older.

Topic 3: Disease transmission and biosecurity practices

• Keywords: transmission, protocol, compliance, PED, practice, animal, grow‐finish, and producer.

Topic 4: Risk factors for disease spread

• Keywords: virus, feed, load, barn, trailer, risk, grow‐finish, and company.

Topic 5: Producer support and biosecurity extension/training

• Keywords: producer, help, operation, extension, and pork.

### 3.4. Sentiment Analysis

Sentiment analysis showed all (100%) of the responses from key informant interviews were categorized as “Positive” regarding swine biosecurity measures. This generally suggests a supportive tone towards biosecurity principles. The analysis approach may, however, overlook subtle or context‐specific expressions that are examined in greater detail in the discussion section.

### 3.5. Thematic Analysis

Thematic analysis identified seven major themes. The identified themes show key patterns about how biosecurity is perceived, practiced, and challenged across different swine operation systems. The identified themes are detailed later and additional quotes selected for each theme are indicated in Table 3.

**Table 3:** Table of themes and representative quotes.

Themes	Representative quotes
Variation in biosecurity practice across farm types	“Sow farms are very locked down. Once you get to the grow‐finish system, that’s where it kind of falls apart. And a lot of that has to do with time and staff,” KI 5
	“To protect sow farms, we’ve utilized most of the science available, along with follow‐up interventions and biosecurity protocols that we audit and try to follow without fail,” KI 8
	“In wean‐to‐finish farms, you’re going in and out frequently, there’s less enforcement of biosecurity…, grow‐finish sites often lack consistent quarantine measures,” KI 3
	“On a sow farm, you have someone monitoring compliance most of the time, but in grow‐finish operations, people often go to sites unsupervised…, Typically, sow production has a higher level of biosecurity than the growing stages, though that’s a generalization. Within each category, you’ll find farms with an extremely high level of biosecurity, including measures to block mechanical transmission, aerosol transmission, and transmission through feed,” KI 8
Human behavior as a barrier and opportunity	There’s often a gap between what people believe they should do and what they actually do,” KI 1
	“People get overworked, and they cut corners because they have too much to do. When farms are short‐staffed, biosecurity takes a backseat,” KI 3
Infrastructure and structural design limitations	“There are cases where even if you want to be compliant, the building doesn’t allow it,” KI 4
Economic constraints and risk‐based decision‐making	“You can’t be fully bio‐secure every day and still survive economically,” KI 4
	“At the end of the day, cost often dictates biosecurity decisions,” KI 5
	“It’s not a lack of knowledge; it’s the financial aspect that poses the greatest challenge,” KI 6
	“A lot of our decisions are based on risk; we’re not going to spend thousands on filtration unless there’s a known threat,” KI 6
	“For a lot of farms, it comes down to money. Even if they want to do it right, they just can’t afford it,” KI 8
Training and communication through a trusted source	“Training is not one‐size‐fits‐all. You’ve got to tailor it; some people need pictures, some need hands‐on. Tools like Glow Germ demonstrations can be effective in creating visual learning experiences and securing buy‐in from farm employees”, KI 5
	“People trust their veterinarian more than some policy they read in a manual. That’s who can drive change,” KI 2
Critical control points for pathogen introduction and spread in swine farms	“Nothing can move disease faster than a truck,” KI 2
	“What I think is probably the largest risk in the industry, and that’s…the packing plants and the cull markets. And like me, we are not going to win the battle. If we don’t put some of that biosecurity improvement on the packer, they can make or break what happens to the industry if a foreign animal disease comes in. You think about it (packing plant!) is like a giant daycare for every disease you could think of.” KI 6
	“Mechanical transmission of disease is the most important way of moving diseases around,” KI 8
Biosecurity achievements of the farmers	“In some sow farms, filtering definitely reduced the frequency of breaks, but over time, as facilities aged and neighborhoods became even more pig‐dense or as viruses changed, some of those gains slipped away,” KI 1
	“…the industry has improved in terms of all‐in, all‐out management.” KI 3
	“One successful approach has been assigning a dedicated biosecurity manager on sow farms. This person oversees biosecurity compliance, monitors supplies coming into the farm, assists with animal movement, and acts as an additional set of eyes on daily operations,” KI 5
	“Compliance is most successful when the process is simple. I would say complexity is the number one reason protocols aren’t followed as intended. Time constraints and the difficulty of execution are the two biggest factors that impact compliance.” Key Informant Six
	“The biggest success I’ve seen in my career has been the implementation of filtration systems and the associated biosecurity measures,” KI 6
	“On the finisher side, any truck that comes on site without pigs is washed beforehand. If we have a health challenge like a fresh PRRS outbreak, we try to strategically time feed delivery based on the health of the animal,” KI 4

Abbreviation: KI, key informant.

### 3.6. Variation in Biosecurity Practice Across Farm Types

According to key informants, biosecurity practices vary significantly according to the type of operation (e.g., sow farms, boar studs, and finishing barns). The biosecurity protocols at sow farms, together with high‐value breeding facilities, remained strict and uniform while grow‐finish farms demonstrated inconsistency and often had gaps in protocol execution. The farm design, staff size and animal movement patterns, and risk perception levels explained these differences as revealed by the following sentiments from key informant eight.

*“Sow farms are very well locked down (“have strict and well-enforced biosecurity protocols”). Once you get to the farrow-finish system, that’s where it kind of falls apart. …And a lot of that has to do with time and staff.”* Key informant eight further elaborated, *“To protect sow farms, we’ve utilized most of the science available, along with follow-up interventions and biosecurity protocols that we audit and try to follow without fail.”*

#### 3.6.1. Human Behavior as a Barrier and Opportunity

All the participants agreed that human elements, particularly employee compliance and understanding, represented both the primary weakness and the most solvable aspect of biosecurity systems. The protocols existed, but human errors occurred because staff members failed to follow them consistently, or they did not recognize risks or failed to understand the reasoning behind the protocols. The level of staffing, staff motivation, and communication methods also influence compliance, as was described by key informant two.

*“You can write the best SOPs, but if the person doesn’t understand why they have to shower, it won’t work.”* As well as key informant one *“It’s not always about having the protocols, it’s about getting people to believe in them [and act on them].”*

#### 3.6.2. Infrastructure and Structural Design Limitations

The layout of numerous farms, especially older or modified ones, lacks the physical infrastructure to support optimal biosecurity measures. The barriers to biosecurity included entry layout problems in barns, lack of functional LoSs, inadequate shower facilities, and limited personnel hygiene and equipment disinfection spaces.

*“Some barns just weren’t built for biosecurity. You can’t easily create a line of separation without gutting the place.”* Key informant seven

#### 3.6.3. Economic Constraints and Risk‐Based Decision‐Making

Financial constraints were a frequently appearing theme in the study when comparing biosecurity practices between farm sizes and ownership types. The informants emphasized that producers must evaluate the expenses of implementing strict biosecurity measures against their perceived level of risk and economic viability. Investments in biosecurity were often prioritized by farmers after a disease outbreak, as stated by key informant 1.

“*If I spend all my money on biosecurity, I’ll go broke; if I spend no money on biosecurity, I’ll go broke. That sums up our dilemma*.”

While key informant two further elaborated on this biosecurity investment dilemma,

“*Air filtration is very expensive, so there is nothing as bad as putting filtration systems on a couple of farms, and all of them break with PRRS*.”

#### 3.6.4. Training and Communication Through a Trusted Source

Participants emphasized the need for regular training and oversight to reinforce and maintain compliance. Key informants noted that learning preferences and language barriers create challenges for understanding the protocol. Informants highlighted that veterinarians and industry leaders serve as trusted sources to effectively deliver biosecurity expectations. An observation that was very well illustrated by key informant 5,

“*Clear communication and translation are critical to ensuring that protocols are properly understood and followed. One of the biggest challenges is overcoming language barriers on farms. Many operations now have a diverse workforce, including international professionals, and unfortunately, I only speak one language fluently*.”

#### 3.6.5. Key Risk Points for Pathogen Introduction and Spread in Swine Farms

The key informants also identified key nodes of biosecurity risk pathways. While the cleanliness of trailer trucks is manageable before transporting wean pigs, it becomes problematic when dealing with large numbers of finisher pigs ready for marketing. Additionally, truckers pose a risk of spreading PEDVs, particularly during heavy winter nights when they might enter the facility, potentially transferring PEDVs onto the farm. Shared equipment in nursing and finishing farms, the use of commercial operators to pump and apply manure, further complicates biosecurity. Furthermore, packing plants and cull markets were identified as critical points in the biosecurity risk pathway; however, these costs are rarely borne by the operators.

*“Now, many use commercial operators to pump and apply manure to fields. That can be risky day or night, since the same equipment may move from site to site, potentially spreading disease. It’s still a challenge,”* quipped key informant one.

#### 3.6.6. Biosecurity Achievements of the Farmers

Key informants stated that many producers have achieved notable success in implementing some biosecurity practices. Many are highly satisfied with their trucking biosecurity measures, which have effectively prevented the entry of PEDV into their farms. Additionally, several farms using filtration systems have avoided PRRS. Some key informants also noted that biosecurity implementation is most successful when the process is simple. Sentiments that were shared across different study participants, as shown:

“*I think we have good trucking biosecurity; that helped us with PEDV*.” Key informant two.

*“Boar stud has not had a PRRS break in nearly 25 years; I attribute this to two key things. The filtration system, which uses HEPA filters, and the development of the semen distribution center, which helped prevent the risk of PRRS spread through transportation.”* Key informant three.

*“Since we’ve focused heavily on transmission through feed, particularly related to PRRS virus, we have shown a significant drop in new PRRS infections in breeding herds that are employing what we would call high levels of biosecurity. This includes not just traditional mechanical and aerosol transmission, but farms that are using some type of feed mitigant to prevent disease transmission through feed.”* Key informant eight.

## 4. Discussion

Guided by our multitheory approach to the exploration of perceptions, practices, major risk pathways, challenges as well as biosecurity achievements in swine farms, the thematic analysis revealed seven interconnected themes which included (1) the differences in biosecurity practices between different farm types, (2) human behavior as both a barrier to and an opportunity for improved biosecurity, (3) infrastructure and structural limitations to biosecurity adoptions, (4) economic constraints and risk‐based decision‐making, (5) the need for training, monitoring, and reliable communication, (6) key risk points for pathogen introduction and spread in swine farms, and (7) biosecurity achievements of the farmers. These same themes were also reflected and identified in content analysis, such as word frequency analysis, n‐gram analysis, word cloud analysis, and topic modeling using Python (version 3.11), indicating convergence between the different qualitative data analysis approaches.

Sentiment analysis showed the attitude of all key informants was positive towards swine biosecurity measures. While this result generally indicates a supportive tone towards biosecurity practices, it is important to note that the algorithm may be limited in detecting nuanced frustrations, ambivalence, or skepticism expressed in the interviews. Such subtleties were, however, better captured/reflected in the thematic coding. We therefore interpret this sentiment analysis output as an indication of a favorable orientation towards biosecurity, rather than a definitive measure of the underlying attitudes.

The first theme revealed differences in biosecurity practices across production types. This was reflected as “animal management,” through topic modeling and reflected in the most frequent bigrams in N‐analysis as sow farm and grow‐finish; this alignment across methods strengthens confidence in the validity of the findings. The key informant stated that biosecurity implementation improves the higher up you go in the production pyramid; sow production systems generally have higher biosecurity standards than grow‐finisher systems. This gradient of biosecurity standards/implementation can be attributed to the genetic value, and therefore, cost of animals in breeding facilities. In addition, the operations maintain low animal movement, which may allow staff to train and implement protocols effectively. Observations consistent with recent studies that highlighted the variation in biosecurity implementation across operation types; sow farms typically had more complete SPS plans than other operation types [1].

On the other hand, the grow‐finish operation was described as the weakest link in the whole system. The grow‐finish operations handle large numbers of animals, and pigs frequently move between them while operating under tight profit margins. The shorter duration of animal stay in finishing operations may lead some to believe these facilities have lower disease risks, as previously reported, where disease risk perception and biosecurity implementation differed by farm structure, operation type, and ownership model [28, 29]. The perception of reduced disease risk in finishing operations may increase the vulnerability of these facilities to disease introduction and spread.

The second theme focused on human behavior. Human factors emerged as among the critical yet modifiable elements in biosecurity systems. Key informant interviews revealed that there are differences in attitudes among personnel, even when they are given uniform biosecurity education; personnel at the management level and those working in the barn may view biosecurity measures differently, given their unique contextual realities within the production system. The term “people/employees” was also among the most frequently discussed words in the content analysis; in word cloud analysis, word frequency analysis, and as a keyword in topic modeling, reinforcing the role of humans in biosecurity implementation.

The informants emphasized that farm employees’ beliefs, motivations, and comprehension levels were more predictive of compliance than the mere presence of biosecurity SOP. Research by Racicot et al. [30] supports this finding by showing that behavioral gaps can make well‐structured biosecurity plans ineffective. As previously reported [31], challenges such as resistance to change, lack of knowledge, and perceived costs can hinder compliance. The informants noted that the employees demonstrated better compliance when they understood the reasons behind the practices and were convinced of their efficacy, which is in line with the concept based on adult learning theory [32]. These barriers can be addressed through education, training, and demonstrations of the economic benefits of biosecurity, as reported by previous studies [9, 31].

The human element in biosecurity systems presents an interesting paradox because farm personnel represent one of the main vulnerabilities in biosecurity by spreading pathogens within farms, or they may provide opportunities for system enhancement by implementing and enforcing biosecurity, as reported in other studies [3]. Like our current study, past research indicates that busy workers in understaffed operations often struggle to uphold biosecurity protocols (6), indicating that biosecurity failures may stem more from structural failures than individual employee carelessness.

Additionally, employee compliance issues can also arise from, abstract nature of disease risk, lack of understanding the connection between their actions and disease prevention, and lack of consistent biosecurity protocols enforcement, which may create confusion in identifying essential versus recommended safety measures. Effective communication strategies that emphasize the rationale behind biosecurity protocols, rather than simply mandating compliance, can improve adherence. Additionally, designing practical and simple protocols within the constraints of daily farm operations may lead to increased consistency in implementation.

Infrastructure and farm design emerged as crucial constraints. Frequent terms such as barn, shower, and LoS in word frequency, word cloud, and LDA analyses further reinforce this theme. According to key informants, older buildings make up half of the production systems, and they pose challenges for implementing biosecurity, primarily due to inefficient design features like shared entry and exit points, which can hinder the establishment of a functional LoS. The key information further revealed that the majority (75%) of old facilities, especially in grow‐finish operations, lack a proper LoS. In contrast, newer buildings typically have improved biosecurity measures and LoS, but may have conceptual weaknesses, such as placing a rendering box at the end of the driveway. Effective biosecurity infrastructure requires specific design elements that prevent the entry and spread of pathogens [33].

The key informants further elaborated that the original building designs and retrofit facilities do not have suitable areas for implementing LoS or controlled access points. Proper implementation of LoS creates a physical and temporal barrier between potentially contaminated and clean areas and thus its absence allows cross‐contamination and pathogen spread through equipment and personnel moving between areas with different biosecurity levels. The study by De Roest et al. [34] demonstrates how facility design determines biosecurity protocol effectiveness through its ability to define “clean‐dirty” transition zones.

Also of note was the lack of sufficient, comfortable, or well‐designed showers, making them inadequate for all workers. Additionally, limited space and supplies for personnel hygiene and equipment disinfection were said to hinder protocol compliance. The key informants further noted that the small space, uncomfortable facilities, and lack of supplies often cause workers to skip or shorten biosecurity steps, especially under time pressure or during bad weather. Upgrading current facilities to meet modern biosecurity standards demands substantial financial investment, especially for small farms, which may not yield quick returns, especially when no disease outbreaks occur or a visible threat is perceived. In addition to the financial constraints, resistance from older swine production workers accustomed to traditional methods also hinders the upgrading of these facilities.

Implementing biosecurity measures on a farm often necessitates a substantial initial financial investment [35]. This investment includes expenses related to training personnel, procuring necessary equipment, establishing appropriate infrastructure, maintaining ongoing operations, and implementing specific biosecurity practices such as vaccination and air filtration systems [34–36]. The informants noted that the value derived from these investments can vary, and some, such as air filtration, can be more challenging to assess. Similarly, the informants stated that trucks are known to pick up and spread PED. However, the current cost–benefit analysis suggests it is cheaper to run a dirty truck than to clean regularly since truck sanitation is costly. Evaluating the economic impact of biosecurity investments is challenging because prevention benefits are not directly visible, and risk assessments are further complicated by variations in disease risks based on location, past disease occurrences, and production density, as reported in previous studies [37, 38].

Our thematic analysis helped show these economic trade‐offs and risk perceptions as central themes influencing biosecurity decision‐making. Even though terms like risk, diseases, PRRS, and PED frequently appeared in the content analysis, the thematic analysis identified specific issues that underline these terms, such as financial constraints and cost–benefit analysis of implementing biosecurity to reduce the risk associated with these diseases. Lack of direct and frequent appearance of terms related to economic factors in the content analysis as central themes suggest that frequency alone may not reflect conceptual importance. This should guide the interpretation of such qualitative data in future studies.

The key informants added that the relationship between farm size and biosecurity investment adds more complexity. Large farms benefit from economies of scale, allowing them to implement comprehensive biosecurity programs at lower costs, but smaller farms cannot afford the fixed costs for advanced systems. For example, investing in dedicated trucks is necessary for effective biosecurity; however, this cost is prohibitive for smallholder farmers. However, bigger operations might also face difficulties maintaining consistent biosecurity measures when managing multiple sites or many employees. These observations concur with previous studies that reported multifaceted and complex barriers to biosecurity implementation [39].

The key informants acknowledged that the PED outbreak was a wake‐up call for the industry, exposing its lack of preparedness for a FAD outbreak. PED prompted a greater focus on feed safety, heightened vigilance in investigating transmission pathways, and increased awareness of the industry’s insufficient biosecurity level. It also helped introduce biosecurity measures, such as feed mitigants, which reduced disease transmission. This shows producers may delay investments in biosecurity until they can measure prevention costs through economic damage caused by a disease outbreak. This finding confirms previous studies that reported better implementation of biosecurity following outbreaks of diseases such as PRRS [11], influenza [40], and in areas of high pig population, may be due to a higher perception of risk of transmission between adjacent farms [41, 42].

The economic approach of “bio‐management” seems to replace total pathogen exclusion as it represents a practical financial solution. However, this reactive method may sound economically rational from the perspective of individual farms, but it does not align with the objectives of the entire industry. Effective biosecurity practices on one farm create potential advantages for nearby farms and the broader industry. However, these benefits are not often accounted for in single‐farm financial assessments. This presents a typical collective action problem because personal economic motivations may not yield optimal industry‐wide biosecurity outcomes, revealing an area where perhaps financial incentives tied to such collective actions may increase compliance.

The study also identified that communication, training, and trust play a crucial role in biosecurity implementation. This thematic category is reinforced by LDA topic modeling, where a closely aligned topic cluster labeled “Producer Support and Biosecurity Training” emerged as one of the most prominent topics in the transcripts. The informants explained that veterinarians and production managers earned higher trust from staff members compared to external trainers, and that training materials needed to be tailored to the language and literacy levels of the employees.

The key informants highlighted that effective biosecurity implementation requires continuous education, monitoring systems, and communication frameworks that ensure biosecurity principles are understood and applied. A previous study also identified a lack of understanding of biosecurity principles and disease transmission among farmers, showing the need for intervention to fill the gap or address misconceptions [3, 30]. All workers who come onsite, including occasional visitors such as vaccination, loadout crews, or repairmen and technicians, must be trained, as they present higher biosecurity risks because of their exposure to multiple operations. This supports experimental and observational studies that have reported people and vehicles as a source of disease introduction and spread between and within farms [3, 43–45].

Key informants highlighted that the success of training programs depends on learning styles, addressing language barriers, and cultural variations within farm workforces. This shows that a single training approach for all workers may not be effective in groups that have different backgrounds requiring different training approaches. These training requirements may present logistical challenges to farm managers in tailoring training programs to address the needs of various groups.

The development of optimal biosecurity plans requires veterinarian or veterinary consultant involvement because they understand farm operations, employee behavior, and local risks. Previous studies also reported that biosecurity protocol development and implementation success depend on establishing trustworthy relationships between all stakeholders (farm veterinarians, veterinary officials, veterinary consultants, farmers, and farm workers) [46]. Veterinarians are considered trusted sources of information and can also help transform complex disease risk information into simple, concise operational recommendations that apply to specific farms and operations, as reported in previous studies [1, 3]. Informants identified swine practitioner networks and the Pork Producers Association as the most effective channels to reach the highest number of producers with training and educational materials.

The informant noted that biosecurity standards tend to decline gradually when oversight is lacking, though they also cautioned that too much monitoring can cause staff to resist. Previous studies showed that effective oversight combines formal auditing, peer review, and self‐monitoring practices to identify which biosecurity measures should be prioritized and implemented to manage disease risk [3, 42, 47]. The effectiveness of communication hinges on the credibility and trustworthiness of the message source. Messages from veterinarians and production managers are more likely to be accepted and acted upon than those from external sources, confirming previous studies [3, 48, 49]. Regular refreshers, updates on disease risks, and recognition of good biosecurity practices have been emphasized to help sustain employee engagement and compliance over time. Previous studies also emphasized the need for regular training and feedback to ensure biosecurity implementation [1, 3].

The study identified multiple high‐risk nodes, such as transportation, manure handling (via shared equipment), and external contacts (packing plants and cull markets) for the introduction and dissemination of the pathogen in swine operations. Truck movement was identified as one of the most important risk factors for the spread of pathogens such as PEDV and PRRSV. The informant added that trucks carrying finisher pigs are not easy to clean and disinfect due to volume and time pressures. Furthermore, they added that the presence of truck drivers in facilities during the winter season increases the risk of mechanical transmission. This concurs with previous studies that reported that contaminated trailers are effective mechanical spreaders for PEDV and PRRSV [43, 50, 51]. Sharing equipment across multiple farms, such as using commercial operators to pump and apply manure, increases cross‐site contamination risks, as manure can harbor and spread pathogens, as previously observed, emphasizing the need for standardized sanitation practices for manure equipment if sharing cannot be avoided [43].

Packing plants and cull markets were also identified as major biosecurity vulnerabilities because they aggregate pigs from different sources, raising the risk of exposure to pathogens and serving as “giant daycares for disease.” Unfortunately, often the costs of biosecurity in this setting are externalized, leading to an industry‐wide vulnerability, an area key informants highlighted as requiring action to address this risk. This observation aligns with a previous study that identified packing plants and animal markets as key nodes for the potential introduction and spread of foreign animal diseases such as ASF virus (ASFV) [1].

Despite all the challenges discussed in implementing and maintaining enhanced biosecurity protocols, the swine industry has achieved notable success in some areas. This success relies on interventions that target critical routes of pathogen entry and spread. Key informants stated that consistent truck sanitation and all‐in, all‐out management helped them limit cross‐contamination. These observations align with previous studies, which identified transport vehicles as important mechanical vectors of PRRSV and other pathogens and highlighted cleaning and disinfection as essential for risk reduction [43, 51].

Another basis of success was attributed to the installation of air filtration systems, especially in sow and boar stud operations. A key informant stated that their boar stud had remained PRRS‐free for 25 years, due to the use of HEPA filtration and semen distribution centers instead of vehicles visiting the farm for collection. Previous studies also reported that the incidence of airborne PRRSV infection in filtered farms was significantly lower than that of farms that do not have filtration systems [52]. Another related study also confirmed that air filtration may cause an 80% reduction in the risk of introduction of new PRRSV [53].

A key informant noted that implementation of basic biosecurity measures, such as requiring workers to wash their hands upon entry, change clothes, wear gloves, and use hand sanitizers before entering barns, helped to reduce PED spread significantly in wean‐to‐finish operations that often used to see nearly 100 PED outbreaks in nurseries. This shows the importance of simple personnel and hand hygiene strategies. The informants revealed that innovative practices such as feed risk mitigation have also been identified to strongly contribute to disease control, especially for PRRSV. The use of feed mitigants to reduce viral transmission risk concurs with previous experimental and field observations indicating feed as a fomite for PRRSV and PEDV [54]. As stated by key informants, timing feed deliveries strategically (delivery to healthy operations first and then to affected operations) during outbreaks is a proactive response they are using to mitigate disease threats.

## 5. Conclusions

Multiple factors influence the successful implementation of biosecurity in swine operations. Identifying barriers to implementing biosecurity practices on farms is essential for developing solutions to overcome these obstacles. While biosecurity remains crucial to swine farm operations, consistent execution is hindered by differences in farm types, infrastructure limitations, financial constraints, and human behaviors. Addressing infrastructure challenges requires investing in essential facilities and integrating biosecurity design into barn structures. Combining infrastructure upgrades with risk‐based, economically sound decisions can help close biosecurity gaps, especially in grow‐finish systems and older facilities. Financial barriers can be alleviated through government assistance or subsidies, and targeted education and training programs can improve knowledge gaps and perceptions about biosecurity among workers. Recognizing high‐risk points, such as exposure to packing plants, emphasizes the need for biosecurity practices that extend beyond farm boundaries and address interconnected risks throughout the supply chain. Successful biosecurity measures, such as maintaining long‐term PRRS‐free boar studs, implementing air filtration, feed mitigation, and employing dedicated biosecurity managers, show that evidence‐based interventions can lead to real improvements. A comprehensive approach that tackles biosecurity barriers and builds on proven successes will strengthen the consistent application of biosecurity practices, resulting in more resilient and healthier swine farms. Ultimately, the future of swine operations requires shifting from reactive to proactive biosecurity strategies and from compliance‐focused to culture‐driven approaches, which involve ongoing monitoring to identify vulnerabilities, along with continuous training and educational efforts. As a cautionary note, this study reflects the perspectives of experienced stakeholders from large commercial swine operations in the Midwest region of the U.S. Therefore, findings may not generalize to other regions or production systems and should be interpreted within this context.

## Author Contributions

Conceptualization: Michael W. Mahero, Maurine C. Chepkwony, Maria Sol Perez Aguirreburualde, and Dennis N. Makau. Data collection: Michael W. Mahero and Dennis N. Makau. Methodology: Michael W. Mahero, Benti D. Gelalcha, Maurine C. Chepkwony, Colin Yoder, and Dennis N. Makau. Data curation: Benti D. Gelalcha, Oluwaseun Akinyede, Moumita Das, Maurine C. Chepkwony, Michael W. Mahero, and Dennis N. Makau. Formal analysis: Benti D. Gelalcha, Michael W. Mahero, and Dennis N. Makau. Writing – original draft preparation: Benti D. Gelalcha. Writing – review and editing: Michael W. Mahero, Benti D. Gelalcha, Maurine C. Chepkwony, Oluwaseun Akinyede, Moumita Das, Andres Perez, and Colin Yoder. Supervision: Michael W. Mahero and Maria Sol Perez Aguirreburualde. Project administration: Michael W. Mahero, Maria Sol Perez Aguirreburualde, and Maurine C. Chepkwony. Funding acquisition: Michael W. Mahero, Andres Perez, Cesar Corzo, and Maria Sol Perez Aguirreburualde.

## Funding

This research was funded by the USDA APHIS National Animal Disease Preparedness and Response Program (NADPRP) (Grant AP21VSSP0000C023).

## Disclosure

The research investigated biosecurity implementation barriers, critical control points, and achievements in U.S. swine operations in the Midwest regions. The funding organization had no role in the design, execution, interpretation, or writing of the study.

## Ethics Statement

This study was conducted in accordance with the Declaration of Helsinki and reviewed by the Institutional Review Board (IRB) of the University of Minnesota (Protocol Number STUDY00012767). A determination was made that this study did not constitute research on human subjects and thus was exempt from requiring IRB approval.

## Consent

Participants were made aware of the voluntary nature of the exercise and consent to conduct the interview sought in the opening statement of the interview guide.

## Conflicts of Interest

The authors declare no conflicts of interest.

## Supporting Information

Additional supporting information can be found online in the Supporting Information section.

## Supporting information


**Supporting Information** Supporting information on completed SRQR checklist and reporting guidelines used during manuscript preparation available online in the supporting information section as S1_SRQR guidelines checklist.

## Data Availability

The qualitative interview transcripts generated and analyzed during this study contain information that could compromise participant privacy and confidentiality. Therefore, they are not publicly available. De‐identified excerpts supporting the findings of this study can be obtained from the corresponding author upon reasonable request.
